# A critique of using the labels *confirmatory* and *exploratory* in modern psychological research

**DOI:** 10.3389/fpsyg.2022.1020770

**Published:** 2022-12-13

**Authors:** Ross Jacobucci

**Affiliations:** Department of Psychology, University of Notre Dame, Notre Dame, IN, United States

**Keywords:** exploratory research, confirmatory research, big data, machine learning, philosophy of science

## Abstract

Psychological science is experiencing a rise in the application of complex statistical models and, simultaneously, a renewed focus on applying research in a confirmatory manner. This presents a fundamental conflict for psychological researchers as more complex forms of modeling necessarily eschew as stringent of theoretical constraints. In this paper, I argue that this is less of a conflict, and more a result of a continued adherence to applying the overly simplistic labels of *exploratory* and *confirmatory*. These terms mask a distinction between exploratory/confirmatory *research practices* and *modeling*. Further, while many researchers recognize that this dichotomous distinction is better represented as a continuum, this only creates additional problems. Finally, I argue that while a focus on preregistration helps clarify the distinction, psychological research would be better off replacing the terms exploratory and confirmatory with additional levels of detail regarding the goals of the study, modeling details, and scientific method.

## Introduction

Psychology has seen a renewed interest in the application of confirmatory research (e.g., Wagenmakers et al., 2012; Scheel et al., 2020), spurred on by the so-called *replication crisis* (e.g., Maxwell et al., 2015). Of the many factors which play a part in the replication crisis, a consistent theme is that researchers take liberty with several steps in proceeding from concept formation to deriving statistical predictions (Scheel et al., 2020). Characteristically, this flexibility in conducting confirmatory research (i.e., researcher degrees of freedom; Simmons et al., 2011) resulted in the label of “exploratory research” or “wonky stats” (Goldacre, 2009; Wagenmakers et al., 2012) along with “non-confirmatory” research (Scheel et al., 2020) for studies that do not follow a strict confirmatory protocol.

For much of the history of psychological research, just using the terms confirmatory and exploratory were justified, as the majority of psychological research was concerned with theory appraisal, explanation, and first generating hypotheses or theory, followed by testing the predictions deriving from it (Hypothetico-Deductive method; detailed further below). However, the advent of big data, and the corresponding algorithms, has shifted a subset of research, resulting in study procedures and aims being less likely to follow default procedures. Fundamentally, psychological research is increasingly incorporating elements that could be construed as exploratory, with this reflecting a natural progression as models grow increasingly complex. Applying the relatively simplistic labels of exploratory and confirmatory to research papers comes with a number of drawbacks. Exploratory research is often devalued,^1^ regardless of the context in which this takes place. As a result, to describe their research paper as confirmatory, researchers often hide any degree of uncertainty as to the theoretical foundations to their research, fail to report any model modifications made, among many other practices (e.g., Simmons et al., 2011; Gelman and Loken, 2014). Further, researchers often use the term exploratory without regard for the context in which the research takes place: exploratory practices in experimental studies (e.g., Franklin, 2005) are quite distinct from atheoretical studies that apply machine learning to large datasets.

I make the case in this paper that using the labels of exploratory and confirmatory applied to studies as a whole, or aims within a study, come with four primary limitations:

- Conflates research practices and modeling.
- The labels are often used in global ways, masking local decisions.
- Are better represented as a continuum as opposed to discrete labels, but results in arbitrary placements.
- Often serve as proxies for more descriptive terms or procedures.

Failing to detail the motivations behind the study, reasoning underlying the procedures, and the large number of decisions made regarding the data and models severely hinders the study’s impact in building a cumulative body of research. While a large number of papers have advocated for increased reporting of *how* a study was conducted (e.g., Depaoli and Van de Schoot, 2017; Aczel et al., 2020), the focus of this paper is on *why* a study was conducted in the manner reported. Prior to discussing these four primary limitations I provide background on the ways in which the terms confirmatory and exploratory have been depicted in psychological research. This is followed by a set of recommendations for moving beyond simply placing studies or study aims into the overly simplistic boxes of exploratory or confirmatory. Instead, I advocate for providing detail on how replication/generalizability was addressed, the form of reasoning used, and orienting the research with respect to explanation, prediction, or description, as well as theory generation, development or appraisal.

### What do the terms *exploratory* and *confirmatory* mean?

Confirmatory research is a hallmark of science. Stating hypotheses, running a study or experiment to test these hypotheses, and then either finding enough evidence to support or fail to support the hypothesis, is an efficient and important cornerstone to this practice. A common view of what constitutes confirmatory research is a series of *a priori* hypotheses followed by developing a research design (experimental in most cases) to test the hypotheses, gathering data, analysis, and concluded with deductive inference (Jaeger and Halliday, 1998). Hypotheses can only be refuted or not refuted, typically assessed using parametric statistics and *p*-values (Null Hypothesis Significance Testing). One prevailing distinction between confirmatory and exploratory research is in the tradeoff between Type I and Type II errors, with confirmatory research favoring low Type I errors, and exploratory research preferring low Type II error (Snedecor and Cochran, 1980).^2^ This can also result in different critical alpha levels being uses in assessing *p*-values, with exploratory research allowing more liberal conclusions (Jaeger and Halliday, 1998).

The most detailed counter to confirmatory research is exploratory data analysis (EDA). While the term EDA has been used in many ways, referring to both research practices and data analysis, the most common meaning refers to the seminal work of Tukey (1977). Tukey (1977) almost exclusively focuses on the use of data visualization to carry out EDA. This can be used in the case of simply exploring data in examining single variables, assessing the assumptions of relatively simple models such as linear regression, or examining the concordance of the actual data and model implied data in complex models (Gelman, 2004).

Going beyond EDA, the definition of exploratory research is most often defined in terms of what it is not, as opposed to what it is. While confirmatory research involves testing hypotheses, exploratory research involves hypothesis generation: “Explicit hypotheses tested with confirmatory research usually do not spring from an intellectual void but instead are gained through exploratory research” (p. S64; Jaeger and Halliday, 1998). This is in line with Good’s (1983) description of EDA as a mechanism to deepen a theory, by looking for holes (residuals).^3^ It is not that exploratory research is devoid of hypotheses, but instead that the hypotheses are often relatively vague and may evolve over the course of experiments or analyses (Kimmelman et al., 2014).

#### Data types

One consistent distinction is in the types of data aligned with each type of modeling, with confirmatory data analysis (CDA) using mostly experimental data, while EDA typically uses observational data (Good, 1983). Though CDA involves specification of hypotheses to directly inform data collection, EDA is often conducted on data that were collected informally, or secondary data analysis. Further, the terms exploratory and confirmatory are almost exclusively contrasted with respect to more traditional data types, with much less description with respect to big data. However, psychological research is increasingly collecting and analyzing new types of data, such as from magnetic resonance imaging, various types of text data, actigraphy data, to name just a few. These new data types present many opportunities for novel types of hypotheses, additional modeling flexibility, along with some challenges to traditional ways of thinking about exploratory and confirmatory research. For instance, can something be exploratory if there is no available confirmatory counterpart? As an example, datasets with more variables than samples require the use of methods such as ridge regression to overcome computational difficulties faced by ordinary least squares. This severely limits the potential for imparting theory into the analysis, as algorithm constraints, not *a priori* theoretical motivations, are required to reduce the dimensionality of the model. In a larger sense, new data types have the potential to further the distinction between theory, variable selection, and the actual models that are tested, further muddying a study’s labeling of confirmatory/exploratory.

To describe this further, we can address the following question: what does confirmatory research look like in the context of text responses? Text data is not unique in the respect of having very few (if no) studies that are confirmatory in nature, as it is more a characteristic of studies that utilize high-dimensional data, as detailed somewhat previously with P > N datasets. Traditional regression models allow researchers to impart theory in the variables used, sequence of models tested, the use of constraints (such as in the form of no relationship constraints in SEM), or the use of interaction terms or testing mediation models, among others. Each of these theoretical characteristics of regression models do not have an analog in text algorithms, or if they do exist, require a great deal of simplification to make the results interpretable (for instance using dictionary-based approaches such as LIWC (Pennebaker et al., 2001) which are based on *a priori* created dictionaries of words, which have clear drawbacks [e.g., Garten et al., 2018]).

In contrast to the use of dictionaries, text data is most often analyzed using relatively complex latent variable models such as latent dirichlet allocation (LDA; Blei et al., 2003) or latent semantic analysis (LSA; Deerwester et al., 1990). This allows researchers to pose hypotheses related to the existence of latent topics common to participants text responses. Relative to psychology research, these are similar in structure to mixture models (LDA) or factor analysis (LSA). However, in contrast to a method such as factor analysis, neither LDA or LSA allow for theory-based constraints as in the form of specifying specific factor loadings. Further, researchers can move beyond the use of LDA and LSA and model the sequence in which words are used with a host of neural network models (e.g., Mikolov et al., 2013). This requires larger amounts of data, but affords modeling the text in a way that is more in line with the way that the words were produced. Using neural networks to model text represents an extremely complex form of modeling, allowing very little theoretical input. In summary, the complexity of text data restricts the degree of theory that can be imposed to into the statistical models, thus meaning analyzing data of this type would exclusively be referred to as exploratory. Ultimately, new types of data necessarily are paired with less theoretical foundation, thus lending themselves to modeling with more to induction than to theory testing (or perhaps more clearly to discovery as opposed to justification, i.e., Reichenbach, 1938; Howard, 2006).

#### Preregistration

In assessing research articles, readers are required to place trust in the authors that the sequence of procedures that was stated in the article mimics what was done in practice. This trust has come in to question spurred on by the replication crisis (e.g., Morawski, 2019), prompting methods such as preregistration to be proposed as a remedy, which has quickly become popular in psychological research (Simmons et al., 2021). Preregistration allows researchers to state the temporal sequence to hypotheses and analyses, thereby increasing the credibility to the statements made in published research.

Using preregistration as a form of validation to better delineate confirmation and exploration may best be summarized in the following: “First, preregistration provides a clear distinction between confirmatory research that uses data to test hypotheses and exploratory research that uses data to generate hypotheses. Mistaking exploratory results for confirmatory tests leads to misplaced confidence in the replicability of reported results” (Nosek and Lindsay, 2018). Additionally, in differentiating between exploration and confirmation, one can go beyond the distinction of whether hypotheses were stated *a priori*, and delineate whether analyses were planned (confirmation) or unplanned (exploration), mimicking the distinction made above between practices and modeling. This does not have to be the case, but is often equated (Nosek et al., 2019).

### Conflating practices and modeling

In the above characterizations of exploratory and confirmatory, there is a conflation between confirmatory vs. exploratory *research practices*, and confirmatory vs. exploratory *modeling* or *data analysis*. While exploratory can refer to EDA, it can simultaneously be taken to mean not specifying *a priori* hypotheses. On the flip side, the term confirmatory can refer to preregistering hypotheses to ensure that they were in fact stated prior to data collection and analysis, or in the case of larger datasets, the use of confirmatory factor analysis to test a hypothesis regarding latent variables. Below, I provide further distinctions of each.

#### Research practices

More recently, almost a consensus has occurred that researchers can no longer be trusted to accurately detail the steps they took in conducting their study (Moore, 2016; van’t Veer and Giner-Sorolla, 2016; Nosek et al., 2018). By not preregistering aspects of study design or analysis, researchers are afforded a degree of flexibility that can compromise the veracity of resultant conclusions. This has been referred to as researcher degrees of freedom (Simmons et al., 2011; Gelman and Loken, 2014), and often manifests itself as multiple comparisons, or “fishing,” and then reporting the best result. Instead, researchers should preregister the study design and analysis plan, among other components of their study. This offers a safeguard against the reporting of exploratory results as if they were confirmatory, namely saying that the hypotheses were stated prior to the analysis results, not the other way around.

One complication in labeling research practices as exploratory is the blurry line between what is termed exploratory, and what is considered questionable research practices (QRPs; Simmons et al., 2011; John et al., 2012). For instance, exploratory is characterized as “where the hypothesis is found in the data” in Wagenmakers et al. (2012), while a number of QRPs revolve around whether descriptions of the results match the order in which the study took place, possibly best exemplified by “claiming to have predicted an unexpected finding (John et al., 2012). Underlying this distinction is the motivation behind the research practice, which can only be assessed through reporting. Thus, without detailed reporting standards, it is easy to conflate exploratory research with QRPs, thus further disadvantaging those that are truly conducting exploratory research.

This leads into a second component of confirmatory research: the data used to confirm hypotheses (test set) must be separate from the data used to generate the hypotheses (train set). Given the small samples sizes inherent in psychological research (i.e., Etz and Vandekerckhove, 2016), this strategy can rarely be fulfilled in practice. This is often referred to as cross-validation and has seen an upsurge of interest in psychology (Koul et al., 2018; de Rooij and Weeda, 2020). To clear up one point of confusion with regard to the term cross-validation, I first need to distinguish two similar, but separate strategies. We can term the strategy of splitting the sample into two separate datasets the validation set approach (e.g., James et al., 2013; Harrell, 2015), also referred to as the *Learn then Test* paradigm (McArdle, 2012). This strategy is often recommended in both psychological and machine learning research but is rarely used due to requiring large initial sample sizes. A second cross-validation strategy involves only using one dataset but repeating the process of splitting the sample into train and test sets, and selecting different subsets of the data for each split. This form of resampling is most commonly conducted using *k* separate partitions of the data (*k*-fold cross-validation) or the repeated use of bootstrap samples. In both *k*-fold and bootstrap sampling, the part of the sample not used to train the model is used to test the fixed model, allowing for a less biased assessment of model fit. It is important to note that most papers that describe cross-validation as a viable strategy to separate exploratory from confirmatory research (e.g., Behrens, 1997; Wagenmakers et al., 2012; Fife and Rodgers, 2022) are referring to the validation set approach rather than *k*-fold cross-validation or bootstrap sampling. While Haig (2005) describes internal validation procedures, such as the bootstrap or *k*-fold cross-validation, as confirmatory procedures, this statement rests on the assumption that the analytic tool being applied has a low propensity to overfit the data, thus the bootstrapped assessment of fit is close to unbiased. In machine learning, the whole sample fit can be extremely positively biased (for example, see Jacobucci et al., 2021), thus internal validation is required (and ideally external validation) to derive a realistic assessment of initial fit, as the within sample fit is often unworthy of examination.

#### Modeling

Specific statistical methods are often labeled as being exploratory or confirmatory, which typically involves the degree of theoretical specification that a model/algorithm affords. On the exploratory side of the spectrum is EDA, machine learning, and exploratory factor analysis, while linear regression, confirmatory factor analysis, and ANOVA are often characterized as being confirmatory. The distinction is often based on the degree of constraints that a statistical method imposes on the data, with these constraints (e.g., linearity or setting specific relationships to be zero) aligning with specific theoretical foundations. For instance, structural equation modeling (of which regression can be seen as a subset of) allows researchers a large degree of flexibility in the type of relationships that can be specified based on theory, and equally as important, which relationships are specified to be non-existent. Further, from a realist perspective, latent variables are defined as real entities, which is difficult to justify from an exploratory (atheoretical) perspective (Rigdon, 2016). In contrast, machine-learning algorithms are often described as atheoretical or exploratory, which can mainly be ascribed to the lack of opportunity afforded researchers to test or impose specific relationships. Instead, relationships are learned from the data.

This may be best exemplified in the context of confirmatory factor analysis. As an example, one can imagine assessing depression, stress, and anxiety with the Depression Anxiety and Stress Scale (DASS-21; Lovibond and Lovibond, 1995). With this, three latent variables would be posed, and in an ideal scenario, a researcher has a fully specified factor model, which mainly involves assigning which observed variables load on which latent variables. A dilemma is faced in the common result of the fit indices indicating some degree of non-optimal fit, which could either be evidenced consistently or inconsistently across multiple indices. Often, this results in researchers searching among the modification indices, making small tweaks here and there to residual covariances, which are typically not reported in the manuscript (Hermida, 2015). Alternatively, if the fit indices are too far away from “good” fit to be salvaged by a handful of *post-hoc* modifications, researchers could return to the “exploratory” phase, often using exploratory factor analysis to reassess either the number of latent variables or which observed variables require cross-loadings. This is one of the few options afforded researchers in this position, as more traditional visualization tools aren’t designed to address lack of misfit indicated by fit indices, and alternative methods such as the use of modification indices have a more general negative reputation (e.g., see MacCallum et al., 1992).

However, newer types of statistical models, particularly those associated with machine learning, are less likely to allow for constraints based on theoretical justification, instead falling more in line with the “throw everything in” approach to data analysis. This is far too simple of a characterization, but one that is generally proffered around by researchers averse to the use of machine learnings methods. The danger in making distinctions such as this is that nothing about the statistics or math is inherently exploratory. Statistical methods are just that, statistical methods. It is how one uses these methods that make them either confirmatory or exploratory. Further, affixing the term confirmatory to a specific statistical method can obfuscate the lack of strong theory (Lilienfeld and Pinto, 2015), giving the researchers a false sense of confidence to the degree of theory imparted in the study^4^.

In examining the labeling conventions based on the degree of constraints the specific statistical methods afford, one quickly runs into contradictions. For instance, EFA actually makes a number of relatively restrictive assumptions, namely that a reflective, not formative model is most appropriate, the relationships are linear, local independence, and researchers can specifically test a hypothesis regarding the number of factors. On the other hand, machine learning algorithms can be used in ways to assess theoretical statements, such as the existence of interactions and/or nonlinearity, fit a linear regression model in the presence of collinearity (using ridge regression), and test new forms of hypotheses (detailed later). Further, the degree of constraint placed on the model or number of parameters does not always align with labeling conventions. For example, lasso regression is often labeled as machine learning despite often having fewer parameters than linear regression. The takeaway point in this discussion is that it is seldom justified to affix the labels of confirmatory or exploratory to specific statistical methods, as almost any method can be used in a confirmatory or exploratory manner (for a similar argument, see McArdle, 1996).

### Consequences

A consequence of labeling the preponderance of methods available as confirmatory induces a feeling of guilt when researchers may not have a concrete hypothesis (McArdle, 2012). Instead, researchers skirt the issue by hiding the exploratory nature of the analysis, and only reporting the best fitting model, or the results and conclusions the arrived at after a considerable degree of fiddling (such as using modification indices without reporting) with the data and models, thus confirming the need for preregistration. Further, the devaluation of exploratory methods/questions (i.e., as exploratory) limits transparent theory generation and imply that researchers should always magically have a rigorous hypothesis to test.

A large percentage of modern research does not fit neatly into the above descriptions of exploratory and confirmatory research for a number of reasons. Further, the descriptions of confirmatory research have seen little application to more recent, complex psychological research studies, thus resulting in a large portion of recent, complex research being labeled as exploratory, despite containing multiple theoretical aspects.

The confusion surrounding the distinction between the terms *confirmatory* and *exploratory* is mainly due to a conflation of two separate questions:

1. How much theory is imparted into various aspects of the study/analysis?
2. What steps have been taken to ensure replicability or generalizability?

Whereas the majority of machine learning studies treat these questions as completely separate (especially with respect to point #2 and the use of cross-validation), as well as being relatively straightforward to answer, these questions are evaluated on a single dimension in much of psychological research. Most often, the recommendations made to address the replication crisis comprise both questions, such as advocating for more concrete theories (e.g., Mansell and Huddy, 2018), not conducting exploratory statistics, and preregistration.

One dimension that specifically muddies the distinction between both questions is the lack of reporting characteristics of many research articles. If researchers do not report what models were tested, it is impossible to determine a clear answer to #1, thus altering what steps should be made for answering #2. As a typical example, if researchers only report a single CFA model that fits well and do not report steps taken conducting EFA, various modifications made to the CFA that were based on modification indices, among others, then a false sense of confidence would be placed into the authors answer to questions #2 by reporting various fit indices that have strong simulation evidence for their ability to assess model fit. This is further discussed below with respect to preregistration.

Part of the confusion regarding the distinction between exploratory and confirmatory concerns the term “hypothesis.” Most accounts describe a hypothesis as a specific, well-formulated statement. Part of the motivation for this may stem from philosophy of science’s fixation on hard sciences, such as physics, where general laws can take mathematical forms. In reality, particularly in psychological research, a hypothesis more often “is nothing but an ebullition of alternative ideas and a pure emotion – consuming speculative curiosity about a certain aspect of the world” (Cattell, 1966). A further problem with the term hypothesis is its generalization from an introductory statistics formulation (i.e., H_0_ = no effect) to complex theoretical formulations. A hypothesis taking the form of a single sentence necessarily denotes some form of reductionism from theory, while a hypothesis matching the degree of theoretical complexity would require, at the very least, a paragraph of formulation. Even with the more recent calls to match theory with mathematical structures in the areas of computational modeling (e.g., Fried, 2020), the degree of complexity necessitates some degree of simplification (DeYoung and Krueger, 2020). This critique of how hypotheses are often specified has consequences for determining the degree of theoretical foundation for studies, as vague hypotheses leave considerable leeway in data analysis.

## Global versus local

In psychological research, the labels of confirmatory/exploratory have been applied to entire studies, or specific aims/hypotheses within a study. Below, I make the case that applying these labels at the global level can mask inconsistencies in the theoretical rationale for decisions at the local level. The number of local decisions that require theoretical justification to follow a truly confirmatory protocol grows almost exponentially as the size of data and number of algorithms considered grows. This can take place with respect to both modeling and the levels of analysis.

### Modeling details

While it is not feasible to describe all the possible aspects that go into a research study, I provide a number of dimensions in Table 1 that are often characteristic of using more complex statistical algorithms, with further detail on what these components look like when based on theory or are atheoretical. The purpose in detailing several dimensions inherent in statistical modeling is to point out how just describing a study as confirmatory or exploratory gives very limited insight into the level (or lack thereof) of theory inherent in each analysis decision.

**Table 1:** Decomposition of multiple study components as to whether the decisions are theory based or non-theory based.

	Theory based	Non-theory based
Algorithm	Algorithm inclusion based on hypothesized relationships in data.	Algorithm inclusion based on convenience or maximizing prediction.
Hyperparameters	Set to be single values or a small set.	Based on software defaults or test a wide range.
Variable inclusion	Each variable is justified.	Variables are chosen based on convenience.
Functional form	Each specified functional form (e.g., linear) should have a theoretical meaning/rationale	Flexibility is inherent in the algorithm to fit a range of functional forms for each relationship
Variable importance/strength	All or a subset of relationships are specified.	Using Ensembles to derive variable importance.
Model choice	Prefer Parsimony.	Prefer Best Fit.

Other researchers have acknowledged the inherent complexity in modern modeling, while advocating for incorporating both preregistration for detailing decisions gone into confirmatory models, along with postregistration for steps taken in conducting follow up, exploratory analyses (Lee et al., 2019). However, this presumes that despite acknowledging that the confirmatory modeling step has a large number of decisions to be made, and flexibility with regard to their choices, the researchers are able to somewhat confidently decide among this myriad of options to formulate the preregistration plan. In contrast, I am advocating for acknowledging which aspects of the modeling procedure are set based on theory, and which there is some degree of uncertainty. In the end, both perspectives could have the exact same outcome, in detailing a preregistration plan with acknowledgement of certain aspects that are tested in the data.

Note that the term hypothesis is not provided in the above table. This is done due to the inherent complexity to modern hypotheses, as they rarely take either a purely theoretical form outside of experimental contexts, or a purely atheoretical form. Instead, I view it as more fruitful to focus on adding additional detail regarding the aspects detailed in Table 1, thus being more concrete in translating hypotheses to aspects detailed in Table 1 and the sections below.

### Level of analysis

**Figure 1:**
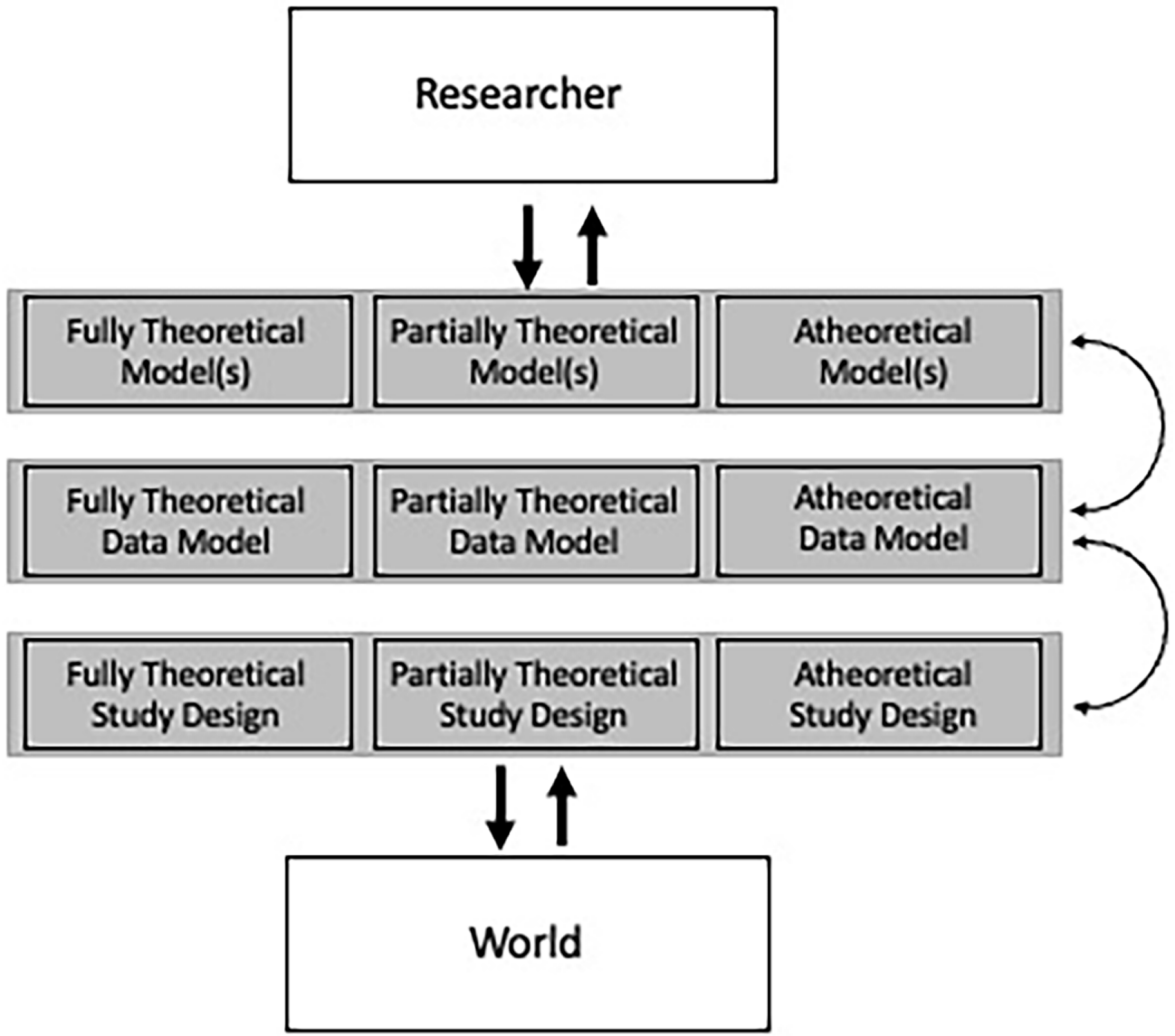
Differentiating various levels of study components based on Suppes (1966) and Kellen (2019).

While larger datasets better afford the fitting of more complex models,^5^ there is not a one-to-one relationship. In fact, larger datasets afford more flexibility in the types of models fit, which can all exist at similar levels of abstraction, or exist across levels. I follow the hierarchy put forth in Kellen (2019; Figure 1), which is based on Suppes (1966), with further elaboration based on this paper’s premise.

In contrast to Kellen (2019), a number of changes were made. First, given that this paper’s focus is not on experimental research, I label this level study design. Further, I partition this based on the degree of theory that went into study design. This is to account for studies that have a theoretical rationale for every variable assessed (fully theoretical), those that have a rationale for a subset of variables (partially theoretical), and those in which data was not directly collected by the researcher, through openly available datasets or other mechanisms. Within this dimension I wish to acknowledge that a majority of modern research studies collect a large number of variables with the aim to use them for several publications.

The second dimension is the data model, which entails the translation of the raw data to that which is used by the modeling procedures. In this, raw variables could be used directly or through summaries of sets of variables depending on the theory, or raw data could be transformed by the models through data-driven dimension reduction. In reality, most research would fall into the partially theoretical box, as many studies only use a small number of variables which are based on summaries, or studies directly specify the use of a subset of variables, while others have a small number of variables of interest, but a multitude of additional variables are “tested” in the form of covariates.

The final dimension concerns the degree of theoretical specification in the modeling stage. Researchers have considerable flexibility in translating a theoretical model to something that conforms to an actual dataset. While this is often criticized (e.g., Yarkoni, 2020), there remains considerable utility to working models that can serve as analogies (Bartha, 2013). In psychological research this may be best exemplified in the debate surrounding the field’s understanding of psychological disorders, with reductionism to neurobiological explanations having historical favoring, but more recent pushback from the network analysis research literature (e.g., Borsboom et al., 2019). These debates seemingly mirror those in other fields, with some sides arguing that including high levels of detail can shed light on theoretical aspects that are under-developed (Fried, 2020), while others see the complexity of real data far outmatching even the most detailed computational model (DeYoung and Krueger, 2020), among many other distinctions.

To provide an example that more concretely distinguishes each level and the degree of theory, we can use a moderation model as an example. Notably, simple moderation models almost always entail theoretical specification of each path, resulting in a path diagram (fully theoretical model). However, there is often a discrepancy between the path diagram and the statistical model used (data model), with a multilevel model adhering closer to the path diagram than the regression model with cross-product terms that is typically fit (Yuan et al., 2014). Finally, while the path diagram contains the names of the variables, researchers have flexibility in whether individual items, summed scores, or factor scores are used to represent each variable.

Both “fully theoretical models” and “atheoretical models” are the subject of most description, possibly best exemplified by mediation models and machine learning algorithms such as neural networks, respectively. However, one could argue that the majority of data models do not fall at either extreme, as most “confirmatory” models have at least parts of the model that were not described in the hypotheses or other parts of the theory formulation. Beyond this, there exist a host of statistical methods that facilitate partially theoretical models. An example is mixture models that are combined with other models, such as growth mixture models (e.g., Ram and Grimm, 2009). In this, a latent growth curve model is specified based on theory, then latent classes are estimated that result in fundamentally different growth trajectories across the classes. This latter model component is not directly based on theory, otherwise researchers could specify a multiple group growth model where the heterogeneity to the growth trajectories is based on observed, not latent, groups.

Atheoretical modeling would take the form of specifying many potential algorithms/models, each of which contain varying degrees of interpretation and propensity to fit the data. One caveat with respect to atheoretical modeling is the common scenario, brought about by increased use of machine learning, where researchers specify a number of algorithms, with the conclusions about the best fitting model having theoretical consequences. This is often conducted in machine learning research, where a linear model fitting better or equal that of a neural network model would lead to the conclusion that linear relationships are sufficient to explain the relations between predictors and outcome.^6^

Part of the goal in detailing the complexity to modern modeling is to encourage researchers to test models/algorithms at varying levels of flexibility. In too much research, researchers pose a model at one level of complexity. The problem with this is that the severity of the hypothesis test is minimal (for instance, see Mayo, 1996). As an example, a large body of clinical applications of machine learning only test a single machine-learning algorithm. The severity of this assessment is significantly bolstered by not just showing that the machine learning algorithm fits the data well, but that it fits the data significantly better than a linear model. This is in contrast to other forms of modeling that are more well established, such as latent growth curve modeling. In this, specifying only a quadratic growth model without assessing the improvement in fit over a linear (or other simpler form) growth model would receive swift criticism.

Lastly, I view the relationship between the theoretical model and data model as underdeveloped in most psychological research (see Ledgerwood, 2018 for similar arguments), which is mainly facilitated by a lack of detail regarding the theoretical model, which is possibly most clearly seen in most studies defaulting to the use of summed scores (of which can often be difficult to describe at a conceptual level, McNeish and Wolf, 2020). While this makes sense if a fully theoretical model is posed that depicts relations between latent constructs, this makes far less sense if the model is less than fully theoretical. As an example, network models pose direct relationships between symptoms, which can often be directly assessed in individual questions, while factor models pose that the latent variables represent coherent summaries of the individual items.

While there is a strong correlation between the descriptors theoretical/atheoretical and confirmatory/exploratory, the important piece is that most studies are multidimensional in nature, and each component deserves detail with respect to the degree of theory imparted. Given that the terms confirmatory/exploratory are most often used to describe studies, I believe that these terms should be replaced with theoretical/atheoretical to denote local details of a study.

## The exploratory–confirmatory gradient

**Figure 2:**
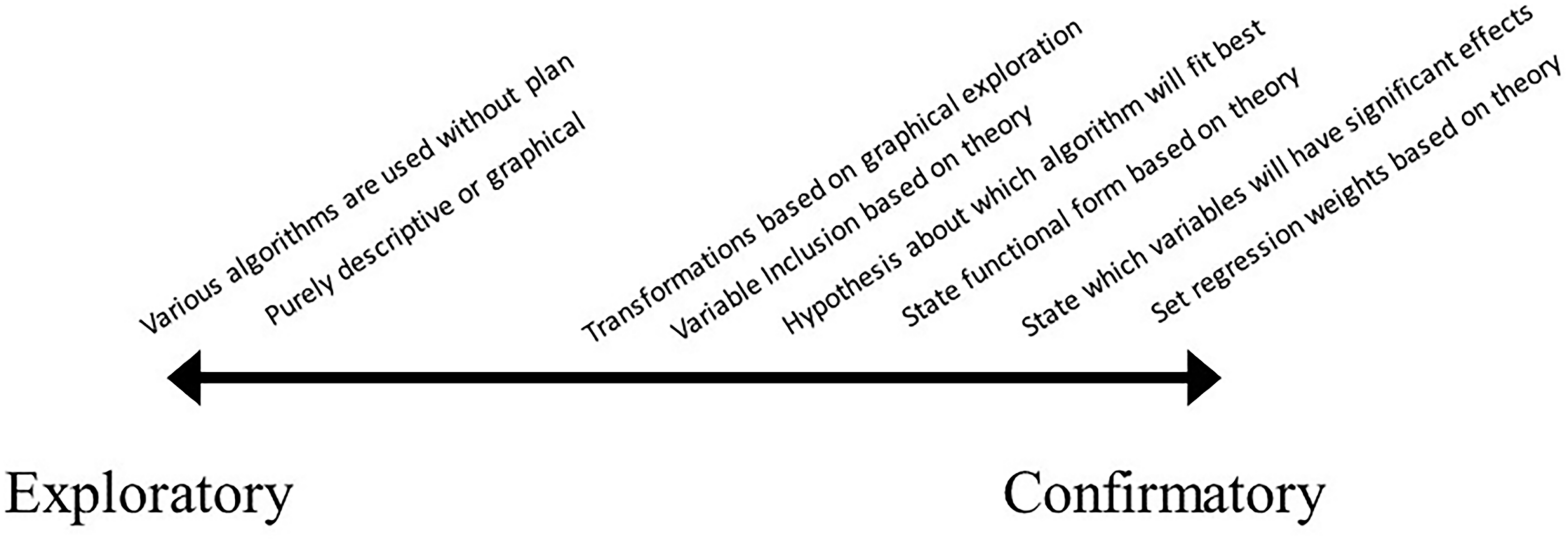
A simplified exploratory–confirmatory gradient. Note that this mainly relates to data analysis, not research practices.

While the labels exploratory and confirmatory are often ascribed in a dichotomous fashion, a large number of researchers have more appropriately seen them as a continuum (e.g., Scheel et al., 2020; Fife and Rodgers, 2022). However, much less detail has been provided on how one identifies where on this continuum a research study falls, let alone individual aspects of a research study. Outside of the label of “rough CDA” to describe research that is mostly confirmatory but also acknowledges some aspects were derived from the data (Tukey, 1977; Fife and Rodgers, 2022), almost no detail has been provided to describe research more accurately. As an example of what this could look like, labels are placed along the continuum from exploratory to confirmatory in Figure 2.

This is in no way meant to be comprehensive, but instead to depict how a select set of scientific practices would likely fall in terms of exploration and confirmation. Most psychological research likely falls in the middle right of the gradient, with some degree of theoretical specification, but stopping short of making specific statements. The far-right hand side of confirmatory corresponds to what Meehl refers to as strong theory, where specific point predictions are made (see bottom of Meehl, 1997, p. 407). The most common conceptualization of exploratory research would fall on the far left of the gradient, where any form of theoretical specification or hypothesis is eschewed in favor of atheoretical modeling.

In this, it is clear to see that the placement of each phrase is relatively arbitrary and could be up for debate. Further, it is easy to imagine research scenarios that have multiple aspects of the design or analysis that occupy different placements on the continuum. In fact, this one-dimensional continuum is only sufficient in the simplest of psychological studies, whereas most modern psychological research entails a large number of decisions, each of which can include varying degrees of theoretical specification (as discussed above). For instance, covariates in a regression could each have been selected based on theory, however, the regression weights were not constrained based on prior research. While this latter specification could seem rather severe, researchers have options such as this to impart strong theoretical ideas (see McArdle, 1996 for further elaboration). Fried (2020) is a more recent discussion of weak versus strong theory, a distinction that mimics the above contrast between exploratory and confirmatory. One could argue that these seemingly parallel lines of contrast are really one and of the same, with researchers hiding behind the “confirmatory” nature of their study to mask what is in reality quite weak theoretical specification.

In the above gradient, there is a clear hierarchical relationship between some of the practices. For instance, detailing which variables are included comes before statements can be made as to which of these variables are likely to have significant effects, and which are not. This further complicates the use of overarching statements of confirmatory or exploratory about the research paper, as the research practices used in the study represent varying points on the continuum.

## Exploratory/confirmatory as proxies

Applying the label confirmatory to describe studies or aims within a study has a significant overlap with whether (1) the study adheres to a Hypothetico-Deductive method, (2) the aims are explanatory, and (3) the study is primarily concerned with theory appraisal. Studies or aims that deviate from these procedures often are required to be labeled as exploratory as they likely follow the inductive process, are descriptive or predictive in nature, or concerned with theory generation. Finally, the terms exploratory/confirmatory have been codified in a sense and replaced by preregistration. We elaborate on each of these dimensions below.

### Method of science

The contrast between exploratory and confirmatory research masks a more fundamental distinction between forms of method, namely between hypothetico-deductive (HD), inductive, and abductive reasoning. I define these terms as (see Fidler et al., 2018 or Haig, 2014 for more detail):

- Inductive: Moving from the specific to the more general. In research, moving from specific observations based on data to the generation of larger theories or principles.
- Hypothetico-Deductive: Moving from general to the more specific. In research, this is generating hypotheses or theory and test the predictions deriving from it.
- Abductive: Commonly referred to as inference to the best explanation. This involves reasoning about hypotheses, models, and theories to explain relevant facts (see Haig, 2005).

The key distinction between the above is whether hypotheses come prior to the data analysis. In contrast to the hypothetico-deductive method, both inductive and abductive can be seen as reasoning from observation (data). While inductive reasoning combines the creation and justification of theories from observation (Haig, 2020), abduction involves the explanation of empirical relations identified in the data through inference to underlying causes.

The distinction of whether theoretical specification/justification comes prior to or after observation mimics prior discussions on distinguishing between exploration and confirmation. Further, the preference for confirmation is mirrored by HD being the most common method used in scientific research (e.g., Mulkay and Gilbert, 1981; Sovacool, 2005). Finally, just as there seems to be a bias against exploratory research, similar things can be said for inductive/abductive research. This is echoed in Fidler et al. (2018): “Part of the solution will be to (a) expand what is considered legitimate scientific activity to include exploratory research that is explicitly presented as exploratory and (b) value the inductive and abductive reasoning supporting this work.”

This procedure in following confirmatory modeling with exploratory analysis could be conceptualized as following what Cattell (1966) termed the Inductive-Hypothetico-Deductive Spiral (Tellegen and Waller, 2008), which could also be said to follow abductive reasoning (Haig, 2005, 2014). With the abductive theory of method, sets of data are analyzed to detect empirical regularities (robust phenomenon), which are then used to develop explanatory theories to explain their existence. This is followed by constructing plausible models through the use of analogy to relevant domains. Finally, if the explanatory theories become well developed, they are then assessed against rival theories with respect to their explanatory value or goodness (e.g., Ylikoski and Kuorikoski, 2010).

While the majority of psychological research operates from a HD perspective, the new forms of data collection and modeling have motivated increased use of either inductive or abductive reasoning. Particularly with large datasets, it becomes increasingly difficult to have a fully formed theory that can be translated into a data model. Further, viewing more complex algorithms such machine learning from the perspective of hypothetico-deductive perspective can lead to unnecessary (terming machine learning as purely exploratory) and strange (classifying machine learning as EDA along with visual displays of residuals; Fife and Rodgers, 2022) formulations.

### Explanation, prediction, description

Similar to how the majority of psychological research has operated from a hypothetico-deductive perspective, thus obviating the necessity of justification, the same could be said for explanatory aims (e.g., Yarkoni and Westfall, 2017). While explanation can be contrasted with description and prediction (Shmueli, 2010; Hamaker et al., 2020; Mõttus et al., 2020), the distinction between description and explanation is often less than clear, and is subject to a researcher’s point of view (Wilkinson, 2014; Yarkoni, 2020). While explanation is concerned with understanding underlying mechanisms.^7^ this is often seen as intimately linked to theory, as in “we typically need to have a theory about what factors may serve as causes,” whereas in descriptive research “we need very little theory to base our research on” (Hamaker et al., 2020, p. 2). Here, we see strong connections to the contrast between exploration and confirmation, as well as between HD and inductive/abductive methods. While there is strong overlap between concepts, describing a study as explanatory clearly orients the reader to the fundamental aim of identifying mechanisms, whereas the concepts of exploration and confirmation are descriptive with respect to theory.

### Theory generation, appraisal, and development

The final dimension is denoting whether a study is primarily concerned with theory generation, development, or appraisal (Haig, 2014). Theory appraisal, quite likely the most common stage of research detailed in psychology publications, is traditionally conducted following HD (e.g., see Locke, 2007), outlining the theory in a hypothesis, then followed by a statistical test. This also corresponds almost directly to previous descriptions of confirmation that rely on hypothesis (theory) testing. Theory development could be seen as either confirmatory or exploratory. Confirmatory if hypotheses are concerned with amendments to specific theory, or exploratory if the theory development is based on following Good’s (1983) description of EDA as a mechanism to deepen a theory. Finally, exploration aligns almost perfectly with the concept of theory generation, which may be best captured by the previously detailed quote: “Explicit hypotheses tested with confirmatory research usually do not spring from an intellectual void but instead are gained through exploratory research” (p. S64; Jaeger and Halliday, 1998).

## Conclusion

Ultimately, the problem with the application of the labels of exploratory/confirmatory can be summarized as an issue in the application of a single dimension solution to multi-dimensional problems. While recent research calls for increased specification on whether the study is exploratory or confirmatory (Wagenmakers et al., 2012; Kimmelman et al., 2014), this will continue to be an overly simple solution. The above sections highlighted deficiencies in their use and how these terms can mask or prevent greater depth in explanation and reporting, particularly in the context of big data. Only the simplest psychological studies could be considered as strictly confirmatory, thus making this exercise futile in generalizing to psychological science broadly.

Each year that goes by results in increased complexity to psychological research, requiring ever more complex levels of decisions made about what variables to collect, which to include in analyses, and what statistical algorithms to use, among many others. Most research cannot and should not be required to have complete theoretical justification for each decision made, as this would severely limit a researcher’s level of flexibility and creativity, not to mention foster deceptive research practices and overstated results. In most contexts the terms confirmatory/exploratory simply refer to whether the Hypothetico-Deductive method was followed across the entire study, or specific hypotheses. Criticisms of the HD approach also apply to the use of exploratory/confirmatory, namely that researchers often feel justified in specifying underdeveloped or vague hypotheses and using non-risky tests (i.e., Fidler et al., 2018).

Instead, the rise of more flexible statistical algorithms has been matched by moves away from more traditional Hypothetico-Deductive research. Instead of pigeonholing these new developments in how research conducted into relatively archaic boxes of exploratory or confirmatory research, I advocate for providing detail on how replication/generalizability was addressed statistically, the form of reasoning used in developing the study procedures, whether explanation, prediction, or description is the primary aim, and finally, what stage of theory generation, development or appraisal the research line is in.

## Data availability statement

The original contributions presented in the study are included in the article/Supplementary materials, further inquiries can be directed to the corresponding author.

## Author contributions

The author confirms being the sole contributor of this work and has approved it for publication.

## Conflict of interest

The author declares that the research was conducted in the absence of any commercial or financial relationships that could be construed as a potential conflict of interest.

## Publisher’s note

All claims expressed in this article are solely those of the authors and do not necessarily represent those of their affiliated organizations, or those of the publisher, the editors and the reviewers. Any product that may be evaluated in this article, or claim that may be made by its manufacturer, is not guaranteed or endorsed by the publisher.

## Footnotes

^1^An extreme example is Lindsay (2015, p. 1828) "Personally, I aim never again to submit for publication a report of a study that was not preregistered. Exploratory work has great and essential value but is typically not appropriate for publication."

^2^This can be equivalently described as exploratory research giving preference to sensitivity, while confirmatory are more concerned with specificity (Kimmelman et al., 2014).

^3^More contemporary research is more likely to utilize additional models or algorithms that have different functional forms or assumptions, capitalizing on developments in computational power (see Lee et al., 2019).

^4^This is why some researchers have proposed replacing the term confirmatory factor analysis with structural factor analysis to better denote that the method places structural constraints on the relationships in the data and is not inherently confirmatory (McArdle, 1996).

^5^I define a model not in the statistical sense, where model is often described based on constraints imposed on probability distributions, yielding distinctions such as exploratory factor analysis being a model while principal components analysis is not (e.g., Kasper and Ünlü, 2013). Further, I contrast this definition with formal models (e.g., Smaldino, 2017; also termed computational models, see Robinaugh et al., 2020), where precise statements are made in the form of equations relating phenomenon. Instead, I use a more general definition, following Bailer-Jones (2009): "a model is an interpretive description of a phenomenon which facilitates access to that phenomenon." There is considerable leeway with the phrase "interpretive description," which leads us to further describe proposed hierarchies to types of models.

^6^Its not nearly this simple, as the measurement of predictors (Jacobson et al., 2021), sample size, among a host of additional factors can influence the comparative fit.

^7^This is an overly simple definition. See Wilkinson (2014) for further detail on various types of explanation.
